# The importance of male body size on sperm uptake and usage, and female fecundity in *Aedes aegypti* and *Aedes albopictus*

**DOI:** 10.1186/s13071-016-1734-8

**Published:** 2016-08-12

**Authors:** Carrie E. De Jesus, Michael H. Reiskind

**Affiliations:** 1 Department of Entomology, North Carolina State University, Box 7613, Raleigh, NC 27695 USA; 2 Present address: Delta Vector Control District, P.O. Box 310, Visalia, CA 93279-0310 USA

**Keywords:** Mating, Container mosquitoes, Accessory gland proteins, Seminal fluid proteins, Male fitness

## Abstract

**Background:**

Adult mosquito density is a critical factor in the transmission of arboviruses by container *Aedes* spp. mosquitoes. Female fecundity drives population growth, and therefore contributes to adult mosquito density. Previous studies have focused on female body size as the major determinant of fecundity, paying little attention to male condition. In this study, we examined the effects of male body size on the abundance of sperm in spermatheca, depletion of sperm over time, and female fecundity.

**Methods:**

We generated males in two size classes using different larval densities, and allowed them to mate with females generated from a moderately dense larval environment. We counted sperm in female spermatheca in a sample of females immediately after mating, then every week for four weeks post-mating. We provided weekly blood meals to females and determined their fecundity over four weeks after the initial blood meal.

**Results:**

We found significantly more sperm in *Aedes albopictus* females than in *Aedes aegypti*, and detected depletion of sperm in *Ae. aegypti*, but not in *Ae. albopictus*. We did not see significant differences in number of sperm in spermathecae in relation to male body size in either species over subsequent gonotrophic cycles. We found a significant effect of male body size on fecundity in *Ae. albopictus*, but not *Ae. aegypti*, with a 46 % increase in fecundity for female *Ae. albopictus* offered four blood meals.

**Conclusions:**

Our results suggest substantial differences in the mating biology of these ecologically similar species and the importance of considering males in understanding female fecundity. The substantial increase in fecundity in *Ae. albopictus* has implications for population growth, estimating vector density, and modeling the transmission of pathogens.

## Background

The density of an arthropod vector is an important component of vectorial capacity and is considered a factor of disease risk in areas of pathogen transmission [1, 2]. Vector density is determined by the presence of a permissive climate, and the longevity and fecundity of females. Determinants of fecundity are critical to estimate disease risk and have been well explored for *Aedes aegypti* L. transmitting dengue, but not for *Aedes albopictus* (Skuse), both of which can be dengue, chikungunya, or Zika virus vectors [3–6].

In mosquito vectors, female body size is strongly correlated with fecundity, with larger females laying more eggs at the first gonotrophic cycle [7–9]. Blood-meal source, age, availability of carbohydrates, and infection status can also affect the number of eggs laid [10–17]. Insemination of the female mosquito is another critical factor for egg-laying, as uninseminated females cannot reproduce. The quality of male mosquitoes has begun to be considered as a potential component of female fitness in *Ae. aegypti* [18, 19], but not in *Ae. albopictus*.

High nutrients, low density and cool temperatures at the larval stage result in large, fecund females and large males for *Aedes* spp. mosquitoes [7, 20]. Although components of male fitness may differ from females, large-sized males seem likely to also be more fit, having higher probability of survival, higher sperm production, and a greater capacity for multiple matings [19, 21, 22]. The effect of male size on the fecundity of individual female mosquitoes has not been directly addressed, although Helinski & Harrington [19] present data showing female *Ae. aegypti* that mate with small males after three previous copulations have reduced fecundity, relative to large males after three copulations. However, when considering just the first mating, there were no significant differences in numbers of eggs laid between females mated with large or small males [19]. Male size does correlate with total numbers of sperm within a male and the number transferred to females [23, 24].

Female spermatheca in *Aedes* spp. mosquitoes have three lobes to store sperm, one larger medial lobe and paired smaller lateral lobes, with usually the medial and one lateral lobe filled after mating [18, 25]. The function of the multiple lobes has not been studied in mosquitoes and is poorly understood in other Diptera [26–28]. One possible function is to sort sperm by selecting one lobe to fill versus another, or to use sperm preferentially from one lobe or another during fertilization [27]. The medial lobe is slightly larger, and has secretory cells that may aid in sperm survival [26]. However, there has been little investigation into sperm storage or use in relation to spermathecal lobes. The usage of different lobes at different points in a female’s life could have important implications for understanding male fitness, particularly if females remate later in life [29]. Male fitness, in turn, can be critical to the success of a variety of sterile male, gene drive, and other novel approaches to mosquito control [18, 30].

In this study, we assessed the importance of male body size on female fecundity and the usage of the spermatheca in releasing sperm. We conducted an experiment using different size-classes of male *Ae. aegypti* and *Ae. albopictus* generated from high and low density larval environments, and allowed them to mate with females generated from a moderate density larval environment. Then we measured female fecundity and sperm count in each spermathecal lobe after each opportunity to blood feed.

## Methods

### Mosquito collection and hatching

*Aedes aegypti* eggs were previously collected in West Palm Beach, (F_8_) Florida [31] and *Ae. albopictus* (F_5_) from Raleigh, North Carolina using oviposition cups. In colony, mosquito larvae were maintained on a diet of koi fish food (Wardy Pond, Pellet, Secaucus, NJ, USA), with 3 pellets per liter of water with approximately 100 larvae/l. Larvae were reared at 27 °C with a 14 L:10D light cycle in incubators. Adults were maintained on human blood from a volunteer (MHR, Approved NCSU Biosafety Committee Protocol 2016-01–0639), given 20 % sucrose *ad libitum*, and allowed to oviposit on seed germination paper. For this experiment, *Ae. aegypti* and *Ae.albopictus* eggs were hatched in 5.69 × 22.81 × 32.99 cm trays (Rubbermaid Egg Keeper, Rubbermaid, Huntersville, NC, USA) filled with 1 l of tap water in a 27 °C incubator for 24 h (Thermo Scientific Precision Incubator 818, ThermoScientific, Marietta, Ohio, USA). Each mosquito species was hatched separately in their own trays.

### Generation of different size classes

We used two larval densities to produce different male body sizes. Trays for generating large male mosquitoes contained 100 larvae and trays for small male mosquitoes contained 250 larvae. We reared females in separate trays with 150 larvae to generate various sized females. Each tray was given 3 pellets of koi fish food in 1 l of tap water, an amount preliminary studies demonstrated to result in density dependent differences in size. We monitored trays daily for the appearance of pupae. We removed pupae immediately and then them separated by sex. We placed pupae into 473 ml plastic cups (Instawares Restaurant Supply, Kennesaw, GA, USA) with a small, plastic 25 ml cup (webstaurantstore.com, Lancaster, PA) with 15 ml of tap water placed at the bottom and checked them daily for the emergence of adults. Adults that emerged in the cups were placed in a climate controlled rearing room at 27 °C with relative humidity at 80 % with a 14:10 light cycle. We provided adults with 20 % sucrose solution. We examined adults in each cup before mating to prevent using individuals from cups with unintended mixed sex adults. Cups with both sexes were discarded. We measured male and female wing lengths as the distance from the alula to the wing tip. Wing length measurements were taken using a dissection scope and measured with a mounted camera and software (Olympus SXZ-LLT, Olympus Cell Sens Standard 1.7.1, MA, USA).

### Mating, blood-feeding and oviposition

We placed 100 adult females in cages (Bug dorm 30 × 30 × 30 cm, Megaview Science, Republic of China) with 100 large or small conspecific males. Females and males were left to mate for 48 h. We sacrificed 10 females to determine insemination rates and sperm counts for each treatment group (all females had been inseminated). We blood-fed 50 females per species and male mating size on a human volunteer (CED) and individually placed each into the same type of cup as the pupae. A piece of seed germination paper (Anchor Paper Co., St. Paul, MN, USA) was wrapped around the edge of the small cup for egg laying. Females were given 1 week to lay eggs before they were offered blood again. We changed egg papers and water before each new blood meal. We counted the number of eggs laid 7 days after a blood meal. Females were kept alive for 1–36 days. Before initial blood feeding and then after every 7 days, 10 live females were sacrificed every 7 days to quantify the sperm present in each spermathecal lobe.

### Spermathecal dissections

Live females were anesthetized with CO_2_ before the spermathecae were dissected. The spermathecae were picked up with a thinned out paint brush tip and rinsed in phosphate buffered saline (PBS, 0.01 M P, 0.0027 M KCl, 0.137 Mm NaCl, pH 7.4; Fisher Scientific Inc.). The 3 spermathecal lobes were then separated from one another. If lobes were torn during the dissection process, those samples were discarded. We did not use dead females for dissections since sperm in the lobes became clumped and could not be dispersed well enough for accurate sperm counts. We placed each individual lobe onto its own glass slide in 7 μl of PBS. We tore apart the lobes with insect pins till a sperm clump was no longer present, as observed under a phase contrast microscope, following the protocol of Perez-Staples et al. [32]. An 18 × 18 mm coverslip was then placed on the slide. We dried the slides for 24 h and examined under a phase-contrast microscope (200×) for sperm count.

### Statistical analysis

We conducted a single run of this experiment. We used a *t*-test to compare male wing lengths of all small and large males (Procedure *T*-TEST, SAS 9.4, SAS Institute Cary, NC, USA). We used a linear mixed model (Procedure GLM, SAS 9.4, SAS Institute, Cary, NC, USA) to determine the effects of male size-class (large, small) and gonotrophic cycle (1–4) on mean sperm count (*n* = 10/ gonotrophic cycle; total and medial and lateral separately) for each species separately. Significant differences were followed by *post-hoc* pairwise comparisons using Bonferonni correction for multiple comparisons. Because sperm counts require the removal of the female, we could not track sperm count through an individual’s lifetime. We did not include sperm from the second lateral lobe examined since none of the *Ae. aegypti* filled it and only 4 *Ae. albopictus* did. Including these sperm did not change the result of any statistical tests examining number of sperm. We compared mean cumulative number of eggs laid before the next blood meal as an effect of male size for each species. We compared the cumulative fecundity before each blood meal separately. We also ran each species as separate analyses. Some females never laid eggs, and were removed from the analysis of egg production, and we assessed differences in having produced a batch of eggs by *χ*^2^-test. One female only produced eggs after several blood meals, and her cumulative fecundity was retained in the data set. Because of a well-established relationship between fecundity and female wing length in mosquitoes [8, 33, 34], we used female wing length as a covariate in all our models examining fecundity. For longevity of females, we used a survival model (Procedure LIFETEST, SAS 9.4) to compare male body size and species using only those females that died naturally (*n* = 78).

## Results

Wing length differed between males reared at high density *versus* low density for both species, with little overlap in sizes (mean ± SEM, *Ae. aegypti*: small males: 1.77 ± 0.02 mm, large males: 2.01 ± 0.01 mm; *t*_(198)_ = -12.39, *P* < 0.0001; *Ae. albopictus*: small males: 1.97 ± 0.03 mm, large males: 2.14 ± 0.01 mm; *t*_(184)_ = -7.24, *P* < 0.0001).

**Fig. 1:**
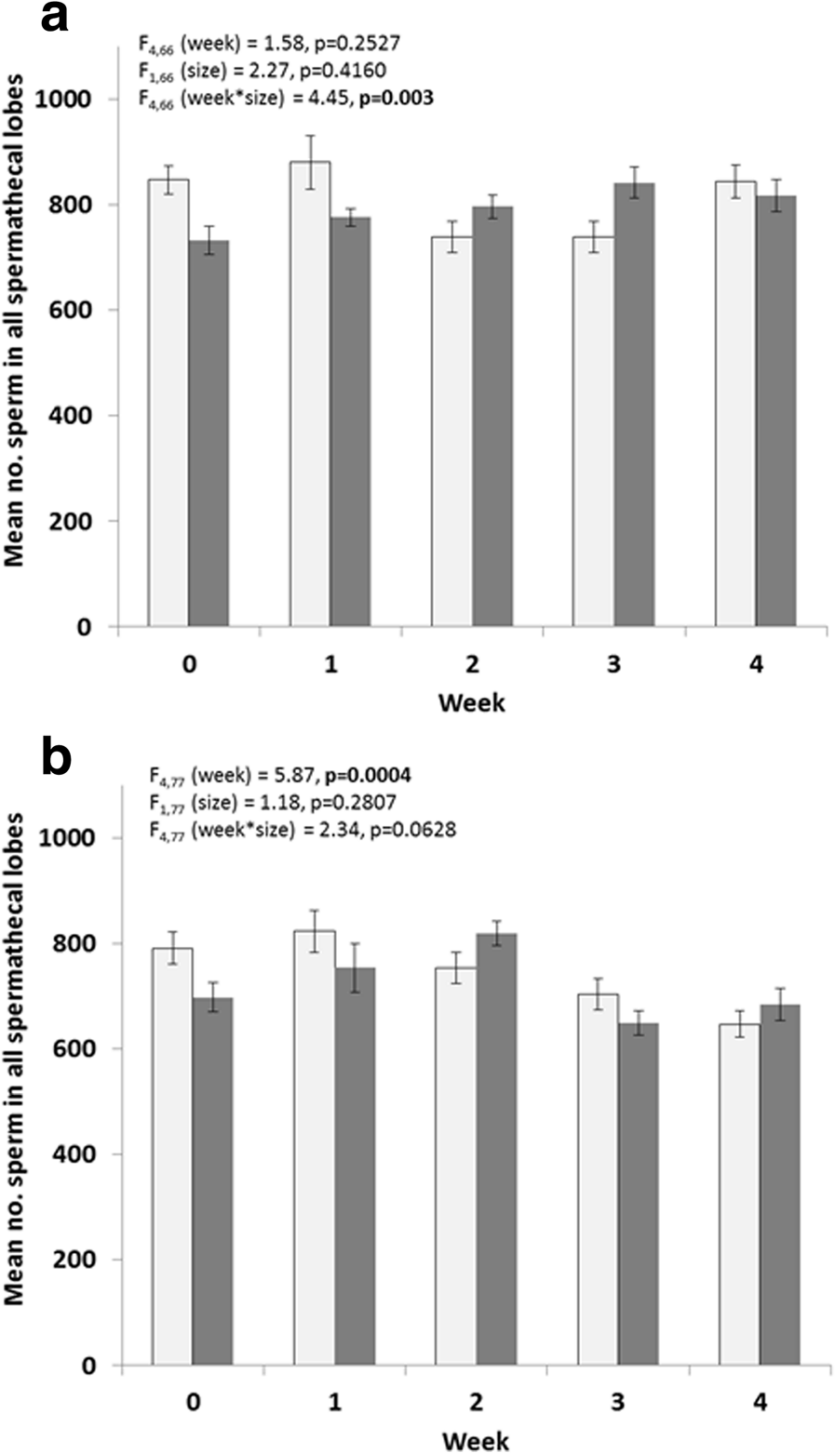
The mean number of sperm in all spermathecal lobes for *Ae. albopictus* **a**, and *Ae. aegypti* **b** from females mated with large males (*light* *grey bars*) and small males (*dark grey* bars) by week. Error bars are ± 1 standard error of the mean (SEM)

There were significantly more sperm found in *Ae. albopictus* spermathecae than in *Ae. aegypti* spermathecae, across all gonotrophic cycles (GLM, *F*_(5,157)_ = 5.21, *P* = 0.0002). There was no correlation between female size and number of sperm for either species (*Ae. aegypti: r*_(75)_ = 0.036, *P* = 0.76; *Ae. albopictus*: *r*_(86)_ = -0.111, *P* = 0.3404). In *Ae. albopictus*, there was a significant interaction between gonotrophic cycle and male size, with a *post-hoc* significant difference between sperm from large males in the first gonotrophic cycle compared to sperm from small males immediately after mating (Model *F*_(9,77)_ = 2.93, *P* = 0.0055; Fig. 1a). The number of sperm in female spermathecae did not differ between large and small males in *Ae. aegypti*, but did decline in gonotrophic cycles 3 and 4 (Model *F*_(9,77)_ = 3.81, *P* = 0.0005; Fig. 1b). There was no interaction between gonotrophic cycle and male size in *Ae. aegypti*.

**Fig. 2:**
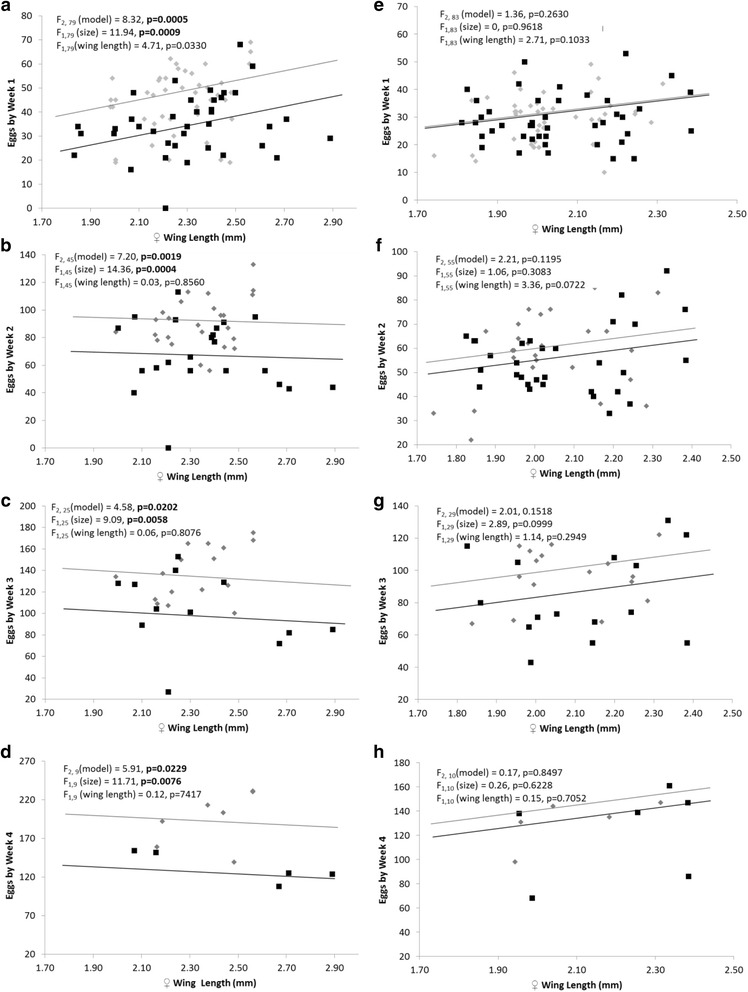
Eggs laid by *Ae. albopictus*, weeks 1–4 (**a**-**d**) and *Ae. aegypti*, weeks 1–4 (**e**-**h**) mated with large males (*grey diamonds*) and small males (*black squares*). Lines show the covariance with female wing length (*grey line*, large males; *black line*, small males)

There were no differences in the number of females that did not produce eggs between treatments (*χ*^2^_3_ = 5.06, *P* = 0.1675). Fecundity before the second blood meal was positively correlated with female wing length for *Ae. albopictus* (*F*_(1,79)_ = 4.71, *P* = 0.033, Fig. 2a), but not for *Ae. aegypti,* nor after subsequent blood meals for either species (Fig. 2b-h). For *Ae. albopictus*, females that had mated with large males produced more eggs after the first and all subsequent blood meals (Fig. 2a-d). When comparing *Ae. albopictus* that made it through four blood meals there was a cumulative difference of 61 eggs between females that mated with large *versus* small males (194.73 *vs* 133.38 eggs, *F*_(1,9)_ = 11.71, *P* = 0.0076, Fig. 2d). There were no significant differences in fecundity in female *Ae. aegypti* mated with large or small males at any point (Figs. 2e-h).

We found no differences in the lifespan female mosquitoes that died naturally as a function of male size, nor any differences between the two species (*χ*^2^_3_ = 5.12, *P* = 0.1630).

## Discussion

Female *Ae. albopictus* had more sperm in their spermathecae than female *Ae. aegypti*. This difference may be a function of differences in size between the species, as *Ae. albopictus* were larger than *Ae. aegypti* in this experiment. There was no correlation between sperm count and female size within each species, and it is impossible to know if sperm count differences between the species are related strictly to size or something intrinsic to the mating biology of each species. We documented depletion of sperm from the spermathecae of *Ae. aegypti*, but not from *Ae. albopictus,* and the mean number of sperm depleted was small. There was considerable variation in sperm counts that may obscure seeing differences. Considering we only allowed females to mate at one point in time, sperm must go down from the initial measurement in nulliparous females to those that have gone through several gonotrophic cycles. However, we were not able to track sperm counts in individuals, and the variation between individuals may have prevented detection of sperm depletion. Variation in sperm count may have also been influenced by polyandry, which is documented at low rates in these species, but we were unable to assess in our study [35–38]. We also only dissected ten or fewer individuals for each treatment at each week, further decreasing our power to detect differences.

We found female *Ae. albopictus* that had the opportunity to mate with larger males produced more eggs, while there was no effect of male body size in *Ae. aegypti*. A possible mechanism of increased female fecundity due to mating with larger males may be driven by the seminal fluid proteins (Sfps) deposited in the female bursa during insemination. These fluids are a complex mixture of sperm and proteins, some of which are conserved and some of which are species-specific, including differences between *Ae. aegypti* and *Ae. albopictus* [39–42]. Ejaculate volume increases with male body size in *Ae. aegypti*, and likely does in *Ae. albopictus*, although we know of no measurements of *Ae. albopictus* ejaculate size [23, 24]. Seminal fluid proteins are known to induce a wide range of female behaviors in insects, including those directly connected to fecundity [43, 44]. These two species are not sibling taxa, and have differences in mating behavior, Sfps components, and the effect of ejaculate in cross mating experiments (e.g. asymmetric mating effects or “satyrization”) [39, 42, 45–47]. Taken together, we hypothesize that larger male *Ae. albopictus* deposit more Sfps than smaller males, and they either change female behavior and/or provide an additional resource for egg development. While *Ae. aegypti* males also likely have size dependent ejaculate volume, the effect of the Sfps on females is more minor [41]. This also fits the observation of asymmetric mating competition in which *Ae. albopictus* males sterilize *Ae. aegypti* females, but the converse is not seen [45, 48]. As we did not assess polyandry in this study, it is also possible that our female mosquitoes mated more than once during the mating period. There are no studies on how polyandry might affect female longevity or fecundity, and the low rates of polyandry make this kind of study difficult. Nevertheless, it is possible that mating with multiple males contributes to the observed differences, and the degree of polyandry may have been influenced by male size. As we only ran a single trial of this experiment it is possible our results are idiosyncratic to something peculiar about our experiment or populations of mosquitoes. Repeating this experiment under further conditions with different genetic stocks of mosquitoes would demonstrate how general this phenomenon is for *Ae. albopictus*.

Our results have important implications for understanding the population dynamics of these two species. In general, population models do not include males, except as larval competitors [4, 6]. However, if male size, as determined by larval environment, has an additional positive effect on female fecundity in *Ae. albopictus*, mathematical population models will need to include male size. We did not explore male fitness *per se*, just the impact of male size of female fecundity. Indeed, as we allowed a small cohort of large or small males to mate with a female, we cannot ascribe the increase in female fecundity to a given male’s size. It is possible this effect is only apparent when there are sufficient males to form a small swarm, for example if the females exposed to the larger males were more polyandrous. Likewise, as we removed females from males after 48 h, the fecundity effects may be different with constant exposure to males, possibly through harassment, as seen in these species and *Drosophila* spp. [49, 50]. If these results hold up to further scrutiny under field conditions, this also suggests that female *Ae. albopictus* should choose larger males, whereas female *Ae. aegypti* may be less discriminating, with consequences for the evolutionary trajectory of males in each species. Future experiments on male size, mating, and female fitness should include a wide variety of mating and larval growth conditions to understand the ecological mechanisms driving our observations.

## Conclusions

In summary, we have demonstrated that for *Ae. albopictus*, male size can have a dramatic impact on fitness. We did not see a similar effect for *Ae. aegypti*, suggesting differences in their mating biology. We have also shown that there is some evidence that sperm are depleted over the lifespan of a female mosquito, but there are still ample sperm to fertilize eggs.

## Abbreviations

*Ae*, *Aedes*

## References

[1] Dye C (1992). The analysis of parasite transmission by bloodsucking insects. Ann Rev Entomol.

[2] Macdonald G (1957). The epidemiology and control of malaria.

[3] Focks D, Haile D, DanielS E, Mount G (1993). Dynamic life table model for *Aedes aegypti* (Diptera, Culicidae) - simulation and validation. J Med Entomol.

[4] Focks DA, Haile DG, Daniels E, Mount GA (1993). Dynamic life table model for *Aedes aegypti* (Diptera, Culcidae) - Analysis of the literature and model development. J Med Entomol.

[5] Focks DA, Daniels E, Haile DG, Keesling JE (1995). A Simulation model of the epidemiology of urban dengue fever - Literature analysis, model development, preliminary validation, and samples of simulation results. Am J Trop Med Hyg.

[6] Magori K, Legros M, Puente ME, Focks DA, Scott TW, Lloyd AL (2009). Skeeter Buster: A stochastic, spatially explicit modeling tool for studying *Aedes aegypti* population replacement and population suppression strategies. Plos Negl Trop Dis.

[7] Briegel H (1990). Metabolic relationship between female body size, reserves, and fecundity of *Aedes aegypti*. J Insect Physiol.

[8] Blackmore MS, Lord CC (2000). The relationship between size and fecundity in *Aedes albopictus*. J Vector Ecol.

[9] Lyimo E, Takken W (1993). Effects of adult body-size on fecundity and the pre-gravid rate of *Anopheles gambiae* females in Tanzania. Med Vet Entomol.

[10] Styer LM, Meola MA, Kramer LD (2007). West Nile virus infection decreases fecundity of *Culex tarsalis* females. J Med Entomol.

[11] Braks MAH, Juliano SA, Lounibos LP (2006). Superior reproductive success on human blood without sugar is not limited to highly anthropophilic mosquito species. Med Vet Entomol.

[12] Styer LM, Minnick SL, Sun AK, Scott TW (2007). Mortality and reproductive dynamics of *Aedes aegypti* (Diptera: Culicidae) fed human blood. Vector-Borne Zoonotic Dis.

[13] Hogg JC, Hurd H (1995). Malaria-induced reduction of fecundity during the first gonotrophic cycle of *Anopheles stephensi* mosquitoes. Med Vet Entomol.

[14] Stone CM, Jackson BT, Foster WA (2012). Effects of plant community composition on the vectorial capacity and fitness of the malaria mosquito *Anopheles gambiae*. Am J Trop Med Hyg.

[15] Bennett G (1970). Influence of blood meal type on fecundity of *Aedes* (*Stegomyia*) *aegypti* L. (Diptera: Culicidae). Can J Zool.

[16] McCann S, Day JF, Allan S, Lord CC (2009). Age modifies the effect of body size on fecundity in *Culex quinquefasciatus* Say (Diptera: Culicidae). J Vector Ecol.

[17] Richards SL, Anderson SL, Yost SA (2012). Effects of blood meal source on the reproduction of *Culex pipiens quinquefasciatus* (Diptera: Culicidae). J Vector Ecol.

[18] Oliva CF, Damiens D, Benedict MQ (2014). Male reproductive biology of *Aedes* mosquitoes. Acta Trop.

[19] Helinski MEH, Harrington LC (2011). Male mating history and body size influence female fecundity and longevity of the dengue vector *Aedes aegypti*. J Med Entomol.

[20] Briegel H, Timmermann SE (2001). *Aedes albopictus* (Diptera: Culicidae): Physiological aspects of development and reproduction. J Med Entomol.

[21] Bader CA, Williams CR (2012). Mating, ovariole number and sperm production of the dengue vector mosquito *Aedes aegypti* (L.) in Australia: broad thermal optima provide the capacity for survival in a changing climate. Physiol Entomol.

[22] Maciel-De-Freitas R, Codeço CT, Lourenço-De-Oliveira R (2007). Body size-associated survival and dispersal rates of *Aedes aegypti* in Rio de Janeiro. Med Vet Entomol.

[23] Ponlawat A, Harrington LC (2007). Age and body size influence male sperm capacity of the dengue vector *Aedes aegypti* (Diptera: Culicidae). J Med Entomol.

[24] Ponlawat A, Harrington LC (2009). Factors associated with male mating success of the dengue vector mosquito, *Aedes aegypti*. Am J Trop Med Hyg.

[25] Christophers SR (1960). *Aëdes aegypti* (L.), the yellow fever mosquito; its life history, bionomics, and structure.

[26] Pascini TV, Ramalho-Ortigao M, Martins GF (2012). Morphological and morphometrical assessment of spermathecae of *Aedes aegypti* females. Mem Inst Oswaldo Cruz.

[27] Ward P (1993). Females influence sperm storage and use in the yellow dung fly *Scathophaga stercoraria* (L.). Behav Ecol Sociobiol.

[28] Fritz A (2004). Sperm storage patterns in singly mated females of the Caribbean fruit fly, *Anastrepha suspensa* (Diptera: Tephritidae). Ann Entomol Soc Am.

[29] Craig G (1967). Mosquitoes - female monogamy induced by male accessory gland substance. Science.

[30] Oliva CF, Damiens D, Vreysen MJB, Lemperiere G, Gilles J (2013). Reproductive strategies of *Aedes albopictus* (Diptera: Culicidae) and implications for the sterile insect technique. PLoS One.

[31] Hopperstad KA, Reiskind MH. Recent changes in the local distribution of yellow fever mosquito, *Aedes aegypti* L. (Diptera: Culicidae) in south Florida, USA. J Med Entomol. 2016 (in press). 10.1093/jme/tjw050 27113103

[32] Perez-Staples D, Harmer AMT, Taylor PW (2007). Sperm storage and utilization in female Queensland fruit flies (*Bactrocera tryoni*). Physiol Entomol.

[33] Armbruster P, Hutchinson RA (2002). Pupal mass and wing length as indicators of fecundity in *Aedes albopictus* and *Aedes geniculatus* (Diptera: Culicidae). J Med Entomol.

[34] Lounibos LP, Suarez S, Menendez Z, Nishimura N, Escher RL, O’Connell SM (2002). Does temperature affect the outcome of larval competition between *Aedes aegypti* and *Aedes albopictus*?. J Vector Ecol.

[35] Richardson JB, Jameson SB, Gloria-Soria A, Wesson DM, Powell J (2015). Evidence of limited polyandry in a natural population of *Aedes aegypti*. Am J Trop Med Hyg.

[36] Helinski MEH, Valerio L, Facchinelli L, Scott TW, Ramsey J, Harrington LC (2012). Evidence of polyandry for *Aedes aegypti* in semifield enclosures. Am J Trop Med Hyg.

[37] Degner EC, Harrington LC (2016). Polyandry depends on postmating time interval in the dengue vector *Aedes aegypti*. Am J Trop Med Hyg.

[38] Boyer S, Toty C, Jacquet M, Lemperiere G, Fontenille D (2012). Evidence of multiple inseminations in the field in *Aedes albopictus*. PLoS One.

[39] Sirot LK, Hardstone MC, Helinski MEH, Ribeiro JMC, Kimura M, Deewatthanawong P (2011). Towards a semen proteome of the dengue vector mosquito: Protein identification and potential functions. Plos Neglect Trop Dis.

[40] Dorus S, Busby SA, Gerike U, Shabanowitz J, Hunt DF, Karr TL (2006). Genomic and functional evolution of the *Drosophila melanogaster* sperm proteome. Nat Genet.

[41] Wasbrough ER, Dorus S, Hester S, Howard-Murkin J, Lilley K, Wilkin E (2010). The *Drosophila melanogaster* sperm proteome-II (DmSP-II). J Proteomics.

[42] Boes KE, Ribeiro JMC, Wong A, Harrington LC, Wolfner MF, Sirot LK (2014). Identification and characterization of seminal fluid proteins in the Asian tiger mosquito, *Aedes albopictus*. Plos Neglect Trop Dis.

[43] Ram KR, Wolfner MF (2007). Seminal influences: *Drosophila* Acps and the molecular interplay between males and females during reproduction. Integ Comp Biol.

[44] Avila FW, Sirot LK, LaFlamme BA, Rubinstein CD, Wolfner MF (2011). Insect seminal fluid proteins: Identification and function. Annu Rev Entomol.

[45] Tripet F, Lounibos LP, Robbins D, Moran J, Nishimura N, Blosser EM (2011). Competitive reduction by satyrization? Evidence for interspecific mating in nature and asymmetric reproductive competition between invasive mosquito vectors. Am J Trop Med Hyg.

[46] Bargielowski I, Blosser E, Lounibos LP (2015). The effects of interspecific courtship on the mating success of *Aedes aegypti* and *Aedes albopictus* (Diptera: Culicidae) males. Ann Entomol Soc Am.

[47] Marcela P, Abu Hassan A, Hamdan A, Dieng H, Kumara TK (2015). Interspecific cross-mating between *Aedes aegypti* and *Aedes albopictus* laboratory strains: Implication of population density on mating behaviors. J Am Mosq Control Assoc.

[48] Bargielowski IE, Lounibos LP, Carrasquilla MC (2013). Evolution of resistance to satyrization through reproductive character displacement in populations of invasive dengue vectors. Proc Nat Acad Sci..

[49] Pitnick S, Garcia-Gonzalez F (2002). Harm to females increases with male body size in *Drosophila melanogaster*. Proc R Soc B.

[50] Soghigian JS, Gibbs K, Stanton A, Kaiser R, Livdahl TP (2014). Sexual harassment and feeding inhibition between two invasive dengue vectors. Env Hlth Insights.

